# Supplementary material for: Oral Vancomycin Prophylaxis for Primary and Secondary Prevention of Clostridioides difficile Infection in Patients Treated with Systemic Antibiotic Therapy: A Systematic Review, Meta-Analysis and Trial Sequential Analysis
Source: Antibiotics (Basel). 2022 Jan 30;11(2):183. doi: 10.3390/antibiotics11020183 (PMC8868369; doi:10.3390/antibiotics11020183)
Supplement: Supplementary file 1 [file antibiotics-11-00183-s001.zip › antibiotics-1555707-supplementary.pdf]

Table S1. PRISMA 2020 (Preferred Reporting Items for Systematic Review and Meta-Analysis) checklist.

Section and Topic	Item #	Checklist item	Reported on page #
**TITLE**			
Title	1	Identify the report as a systematic review.	1
**ABSTRACT**			
Abstract	2	See the PRISMA 2020 for Abstracts checklist.	1
**INTRODUCTION**			
Rationale	3	Describe the rationale for the review in the context of existing knowledge.	2
Objectives	4	Provide an explicit statement of the objective(s) or question(s) the review addresses.	2
**METHODS**			
Eligibility criteria	5	Specify the inclusion and exclusion criteria for the review and how studies were grouped for the syntheses.	3-5
Information sources	6	Specify all databases, registers, websites, organisations, reference lists and other sources searched or consulted to identify studies. Specify the date when each source was last searched or consulted.	3-5, Figure 1
Search strategy	7	Present the full search strategies for all databases, registers and websites, including any filters and limits used.	3-5, Table S2
Selection process	8	Specify the methods used to decide whether a study met the inclusion criteria of the review, including how many reviewers screened each record and each report retrieved, whether they worked independently, and if applicable, details of automation tools used in the process.	3-5
Data collection process	9	Specify the methods used to collect data from reports, including how many reviewers collected data from each report, whether they worked independently, any processes for obtaining or confirming data from study investigators, and if applicable, details of automation tools used in the process.	3-5
Data items	10a	List and define all outcomes for which data were sought. Specify whether all results that were compatible with each outcome domain in each study were sought (e.g. for all measures, time points, analyses), and if not, the methods used to decide which results to collect.	3-5
	10b	List and define all other variables for which data were sought (e.g. participant and intervention characteristics, funding sources). Describe any assumptions made about any missing or unclear information.	3-5
Study risk of bias assessment	11	Specify the methods used to assess risk of bias in the included studies, including details of the tool(s) used, how many reviewers assessed each study and whether they worked independently, and if applicable, details of automation tools used in the process.	3-5
Effect measures	12	Specify for each outcome the effect measure(s) (e.g. risk ratio, mean difference) used in the synthesis or presentation of results.	3-5
Synthesis methods	13a	Describe the processes used to decide which studies were eligible for each synthesis (e.g. tabulating the study intervention characteristics and comparing against the planned groups for each synthesis (item #5)).	3-5
	13b	Describe any methods required to prepare the data for presentation or synthesis, such as handling of missing summary statistics, or data conversions.	3-5
	13c	Describe any methods used to tabulate or visually display results of individual studies and syntheses.	3-5
	13d	Describe any methods used to synthesize results and provide a rationale for the choice(s). If meta-analysis was performed, describe the model(s), method(s) to identify the presence and extent of statistical heterogeneity, and software package(s) used.	3-5
	13e	Describe any methods used to explore possible causes of heterogeneity among study results (e.g. subgroup analysis, meta-regression).	3-5
	13f	Describe any sensitivity analyses conducted to assess robustness of the synthesized results.	3-5
Reporting bias assessment	14	Describe any methods used to assess risk of bias due to missing results in a synthesis (arising from reporting biases).	3-5
Certainty	15	Describe any methods used to assess certainty (or confidence) in the body of evidence for an	6

Section and Topic	Item #	Checklist item	Reported on page #
assessment		outcome.	
**RESULTS**			
Study selection	16a	Describe the results of the search and selection process, from the number of records identified in the search to the number of studies included in the review, ideally using a flow diagram.	6-7, Figure 1
	16b	Cite studies that might appear to meet the inclusion criteria, but which were excluded, and explain why they were excluded.	N/A
Study characteristics	17	Cite each included study and present its characteristics.	Table 1
Risk of bias in studies	18	Present assessments of risk of bias for each included study.	17, Table S4
Results of individual studies	19	For all outcomes, present, for each study: (a) summary statistics for each group (where appropriate) and (b) an effect estimate and its precision (e.g. confidence/credible interval), ideally using structured tables or plots.	Figures 2-5, Table 2
Results of syntheses	20a	For each synthesis, briefly summarise the characteristics and risk of bias among contributing studies.	11-15, Figures 2-5
	20b	Present results of all statistical syntheses conducted. If meta-analysis was done, present for each the summary estimate and its precision (e.g. confidence/credible interval) and measures of statistical heterogeneity. If comparing groups, describe the direction of the effect.	10-17, Figures 2-5
	20c	Present results of all investigations of possible causes of heterogeneity among study results.	11-15
	20d	Present results of all sensitivity analyses conducted to assess the robustness of the synthesized results.	15-16, Figures S5-S8
Reporting biases	21	Present assessments of risk of bias due to missing results (arising from reporting biases) for each synthesis assessed.	17, Table S5
Certainty of evidence	22	Present assessments of certainty (or confidence) in the body of evidence for each outcome assessed.	17, Table S5
**DISCUSSION**			
Discussion	23a	Provide a general interpretation of the results in the context of other evidence.	18-20
	23b	Discuss any limitations of the evidence included in the review.	18-20
	23c	Discuss any limitations of the review processes used.	18-20
	23d	Discuss implications of the results for practice, policy, and future research.	18-20
**OTHER INFORMATION**			
Registration and protocol	24a	Provide registration information for the review, including register name and registration number, or state that the review was not registered.	2
	24b	Indicate where the review protocol can be accessed, or state that a protocol was not prepared.	2
	24c	Describe and explain any amendments to information provided at registration or in the protocol.	3
Support	25	Describe sources of financial or non-financial support for the review, and the role of the funders or sponsors in the review.	20
Competing interests	26	Declare any competing interests of review authors.	20
Availability of data, code and other materials	27	Report which of the following are publicly available and where they can be found: template data collection forms; data extracted from included studies; data used for all analyses; analytic code; any other materials used in the review.	20

From: Page MJ, McKenzie JE, Bossuyt PM, Boutron I, Hoffmann TC, Mulrow CD, et al. The PRISMA 2020 statement: an updated guideline for reporting systematic reviews. BMJ 2021; 372 :n71. doi: 10.1136/bmj.n71

Table S2. Search strategy through electronic databases**.**

**PubMed**	("difficile"[Text Word] OR "clostridium"[Text Word] OR "clostridioides"[Text Word]) AND ("secondary"[Text Word] OR "prevention"[Text Word] OR "recurrence"[Text Word] OR "relapse"[Text Word]) AND "vancomycin"[Text Word] AND "oral"[Text Word])
**EMBASE**	vancomycin:ti,ab,kw AND oral:ti,ab,kw AND (prevention:ti,ab,kw OR prophylaxis:ti,ab,kw OR recurrence:ti,ab,kw OR relapse:ti,ab,kw) AND (clostridioides:ti,ab,kw OR clostridium:ti,ab,kw OR difficile:ti,ab,kw)

Table S3. Variables taken into account to yield adjusted effects sizes (related to OVP efficacy/effectiveness) in the studies providing multivariable analysis.

**Study**	**Variables**
Carignan A et al. [33]	age; number of previous CDI episodes
Caroff DA et al. [38]	age; Elixhauser score; antibiotic risk class; days since most recent positive *Clostridioides difficile* test; number of prior positive *C. difficile* tests; ICU admission
Morrisette T et al. [41]	age; weight; height; sex; race; setting of CDI onset; hematologic malignancy; use of carbapenems; use of fluoroquinolones; duration of high-risk antibiotics after CDI diagnosis
Bao H et al. [43]	recent hospitalization within 30 days; duration of SAT; previous receipt of high-risk antibiotics

Abbreviations: CDI, *Clostridioides difficile* infection; ICU, intensive care unit; OVP: oral vancomycin prophylaxis; SAT: systemic antibiotic therapy.

Figure S1. Meta-analysis regarding the association of OVP with CDI prevention in the setting of secondary prophylaxis, stratifying according to the number of previous CDI episodes.

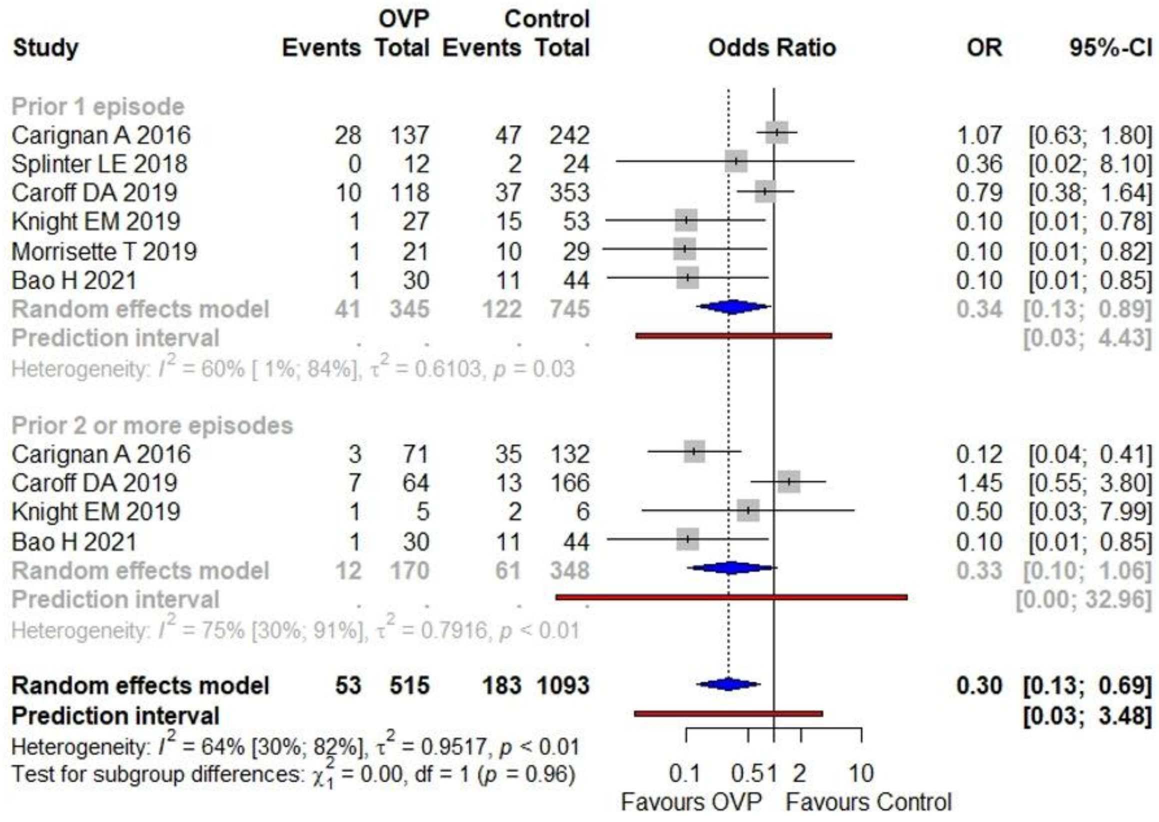

Abbreviations: CDI, *Clostridioides difficile* infection; OR, odds ratio; OVP: oral vancomycin prophylaxis; 95%-CI, confidence intervals at 95%.

Vertical line indicates 'no difference' point between the two options. Squares represent odds ratios. Diamonds represent pooled odds ratios for all studies. Horizontal lines represent 95% CI.

Figure S2. Meta-regression plot of the impact of OVP days on CDI occurrence (considering studies both of primary and secondary prophylaxis) according to a GLMM.

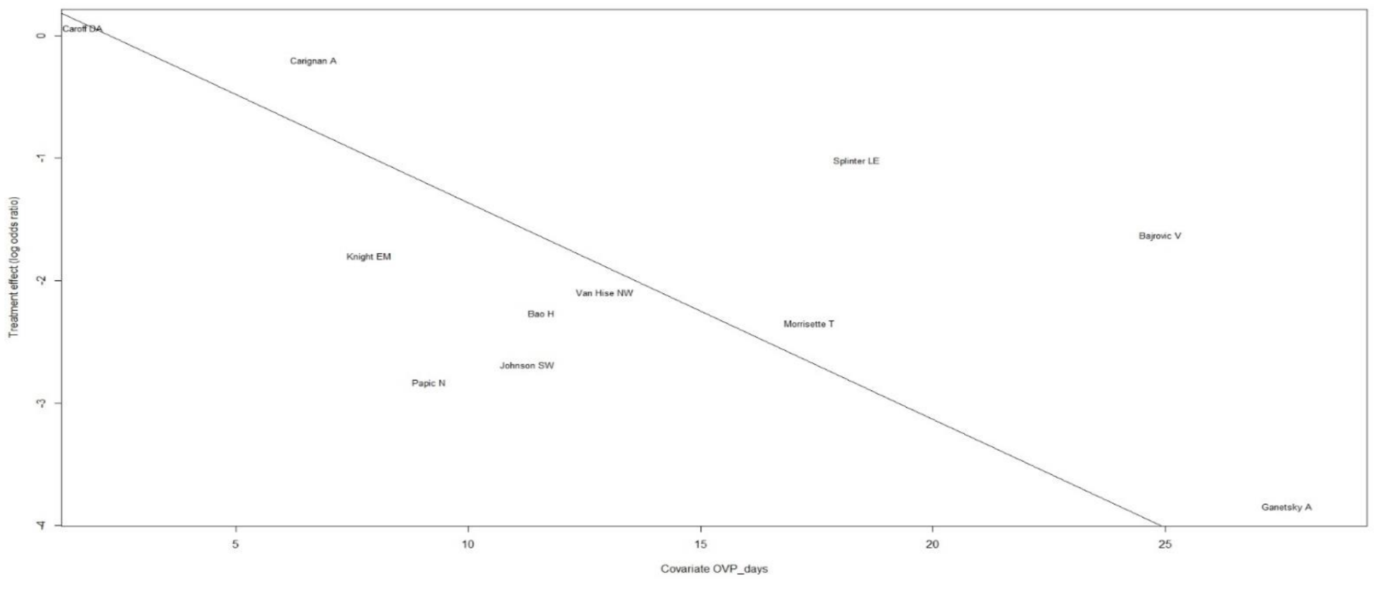

Abbreviations: CDI, *Clostridioides difficile* infection; GLMM, generalized linear mixed models; OVP: oral vancomycin prophylaxis.

Figure S3. Meta-analysis regarding the association of OVP with CDI prevention, overall and across the principal subgroups, according to a Mantel–Haenszel (MH) method without continuity correction.

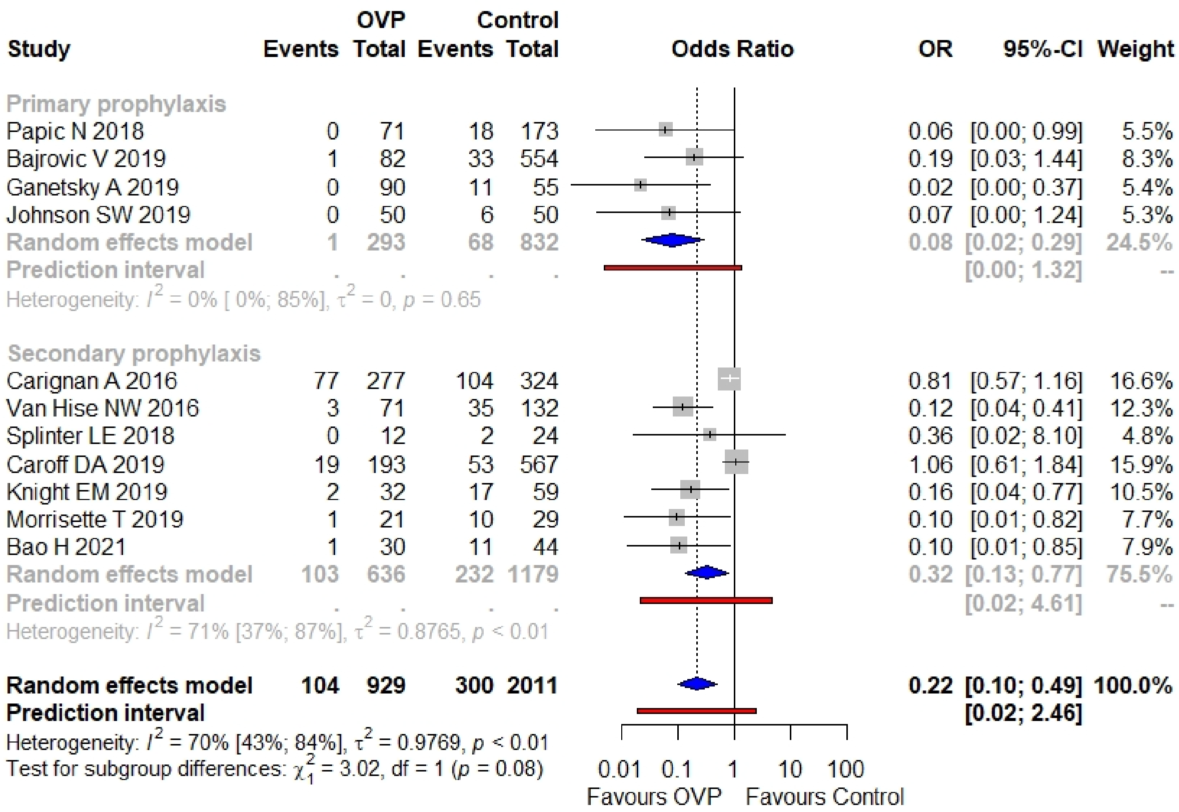

Abbreviations: CDI, *Clostridioides difficile* infection; OR, odds ratio; OVP: oral vancomycin prophylaxis; 95%-CI, confidence intervals at 95%.

Vertical line indicates 'no difference' point between the two options. Squares represent odds ratios. Diamonds represent pooled odds ratios for all studies. Horizontal lines represent 95% CI.

Figure S4. E-value plot concerning primary outcome analysis.

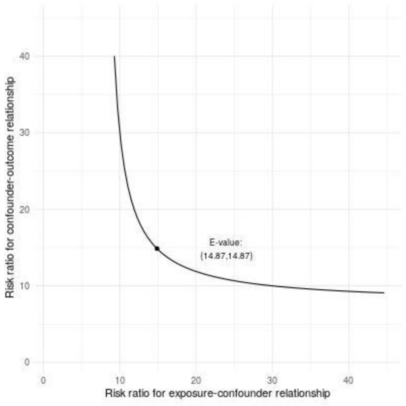

Each point along the curve defines a joint relationship between the two sensitivity parameters that could potentially explain away the estimated effect. If one of the two parameters is smaller than the E-value, the other must be larger, as defined by the plotted curve.

Figure S5. Influential plot visualizing the summary effect sizes and heterogeneity values (I²) for meta-analyses without the study named in each row.

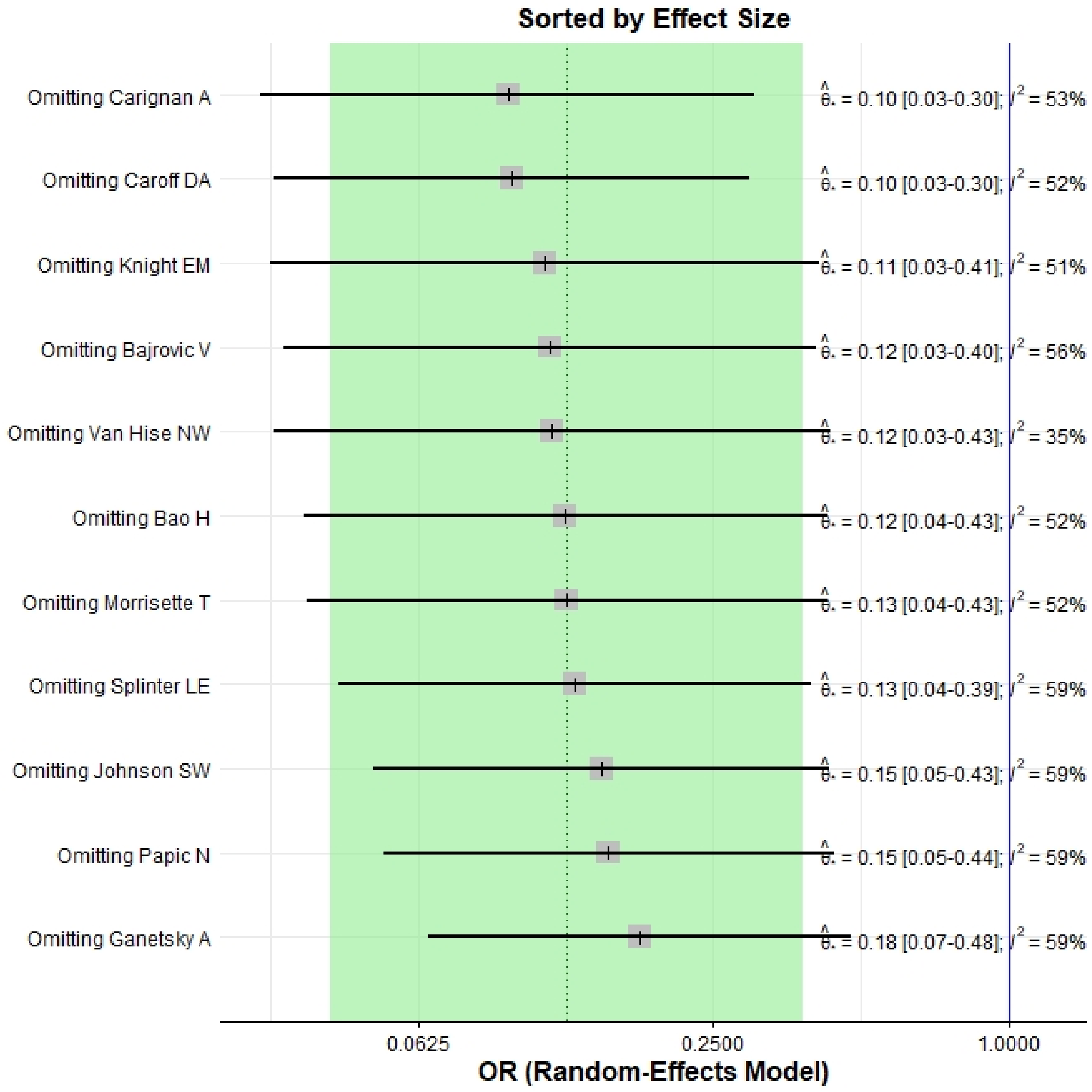

Figure S6. Baujat plot, depicting the contribution of each study to the overall heterogeneity (as measured by Cochran's Q) on the horizontal axis, and its influence on the pooled effect size on the vertical axis.

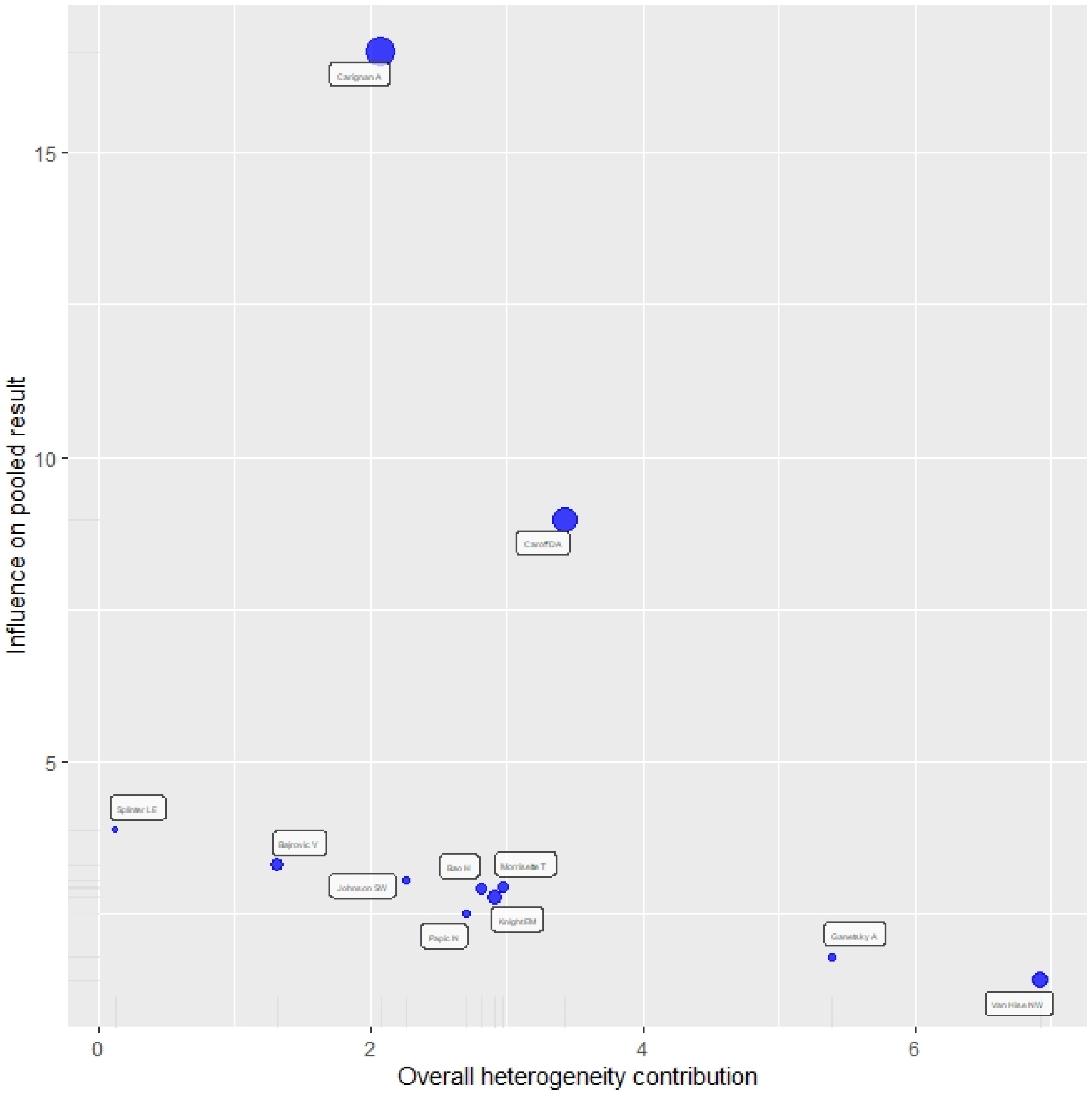

Figure S7. Graphic Display of Heterogeneity (GOSH) plot analysis with related diagnostics to identify potential outliers.

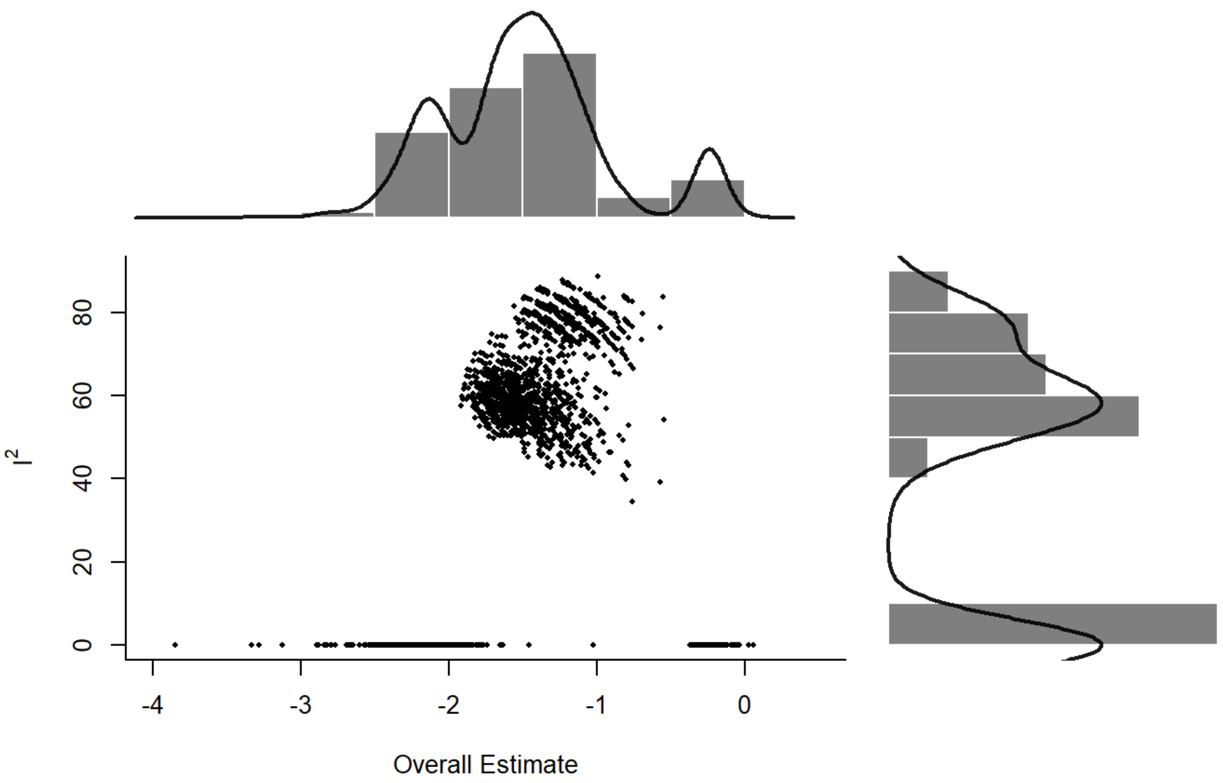

GOSH Diagnostics
================================

- Number of K-means clusters detected: 2
- Number of DBSCAN clusters detected: 3
- Number of GMM clusters detected: 2

Identification of potential outliers

- K-means: Study 1
- DBSCAN: Study 1, Study 6, Study 7
- Gaussian Mixture Model: Study 1, Study 6, Study 7

Study 1 = Carignan et al. 2016
Study 6 = Caroff et al. 2019
Study 7 = Ganetsky et al. 2019

Figure S8. Meta-analysis regarding the association of OVP with CDI prevention by excluding influential studies detected by GOSH diagnostics.

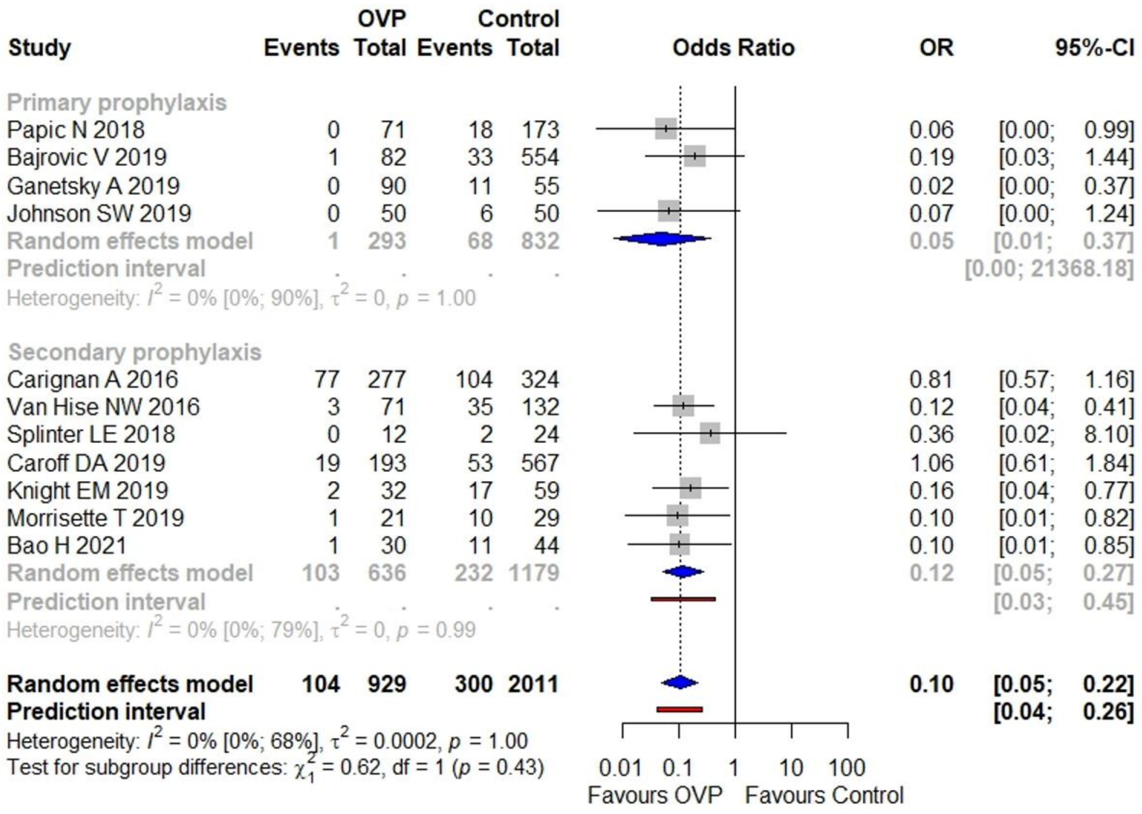

Abbreviations: CDI, *Clostridioides difficile* infection; GOSH, Graphic Display of Heterogeneity; OR, odds ratio; OVP: oral vancomycin prophylaxis; 95%-CI, confidence intervals at 95%.

Vertical line indicates ‘no difference’ point between the two options. Squares represent odds ratios. Diamonds represent pooled odds ratios for all studies. Horizontal lines represent 95% CI.

Figure S9. Meta-regression bubble plot of the impact of OVP days on CDI occurrence (considering studies both of primary and secondary prophylaxis) according to a Mantel–Haenszel (MH) method without continuity correction.

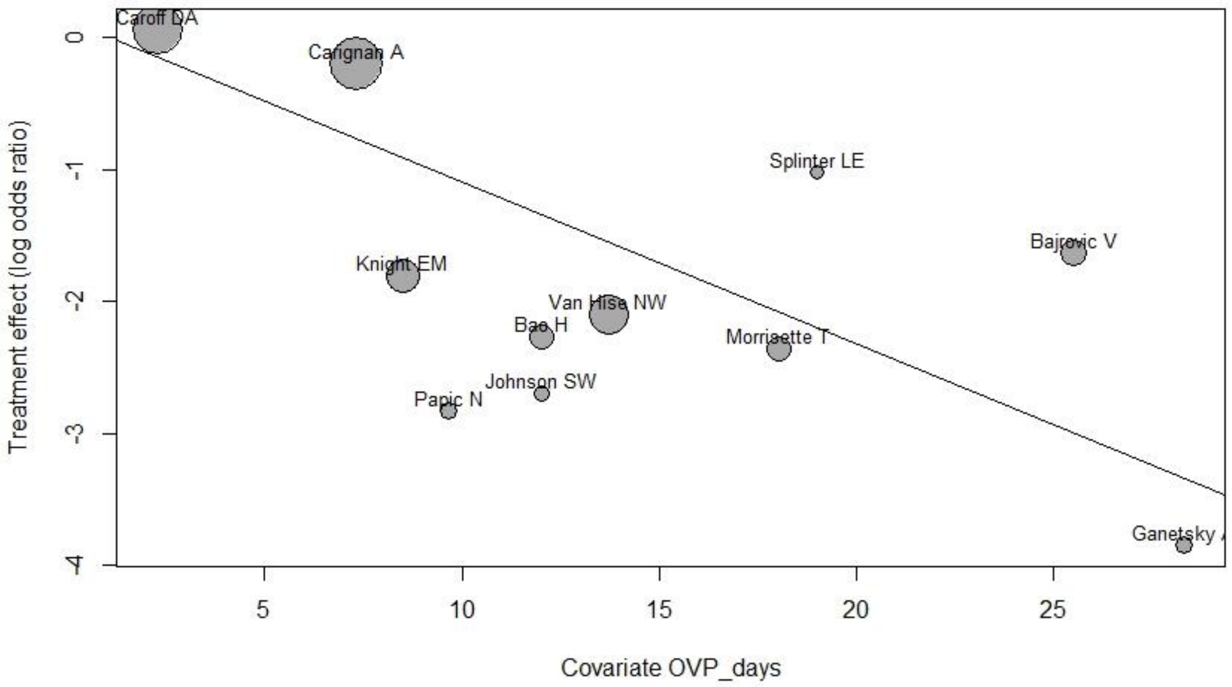

Abbreviations: CDI, *Clostridioides difficile* infection; MH, Mantel–Haenszel; OVP: oral vancomycin prophylaxis.

Figure S10. Contour-enhanced funnel plot.

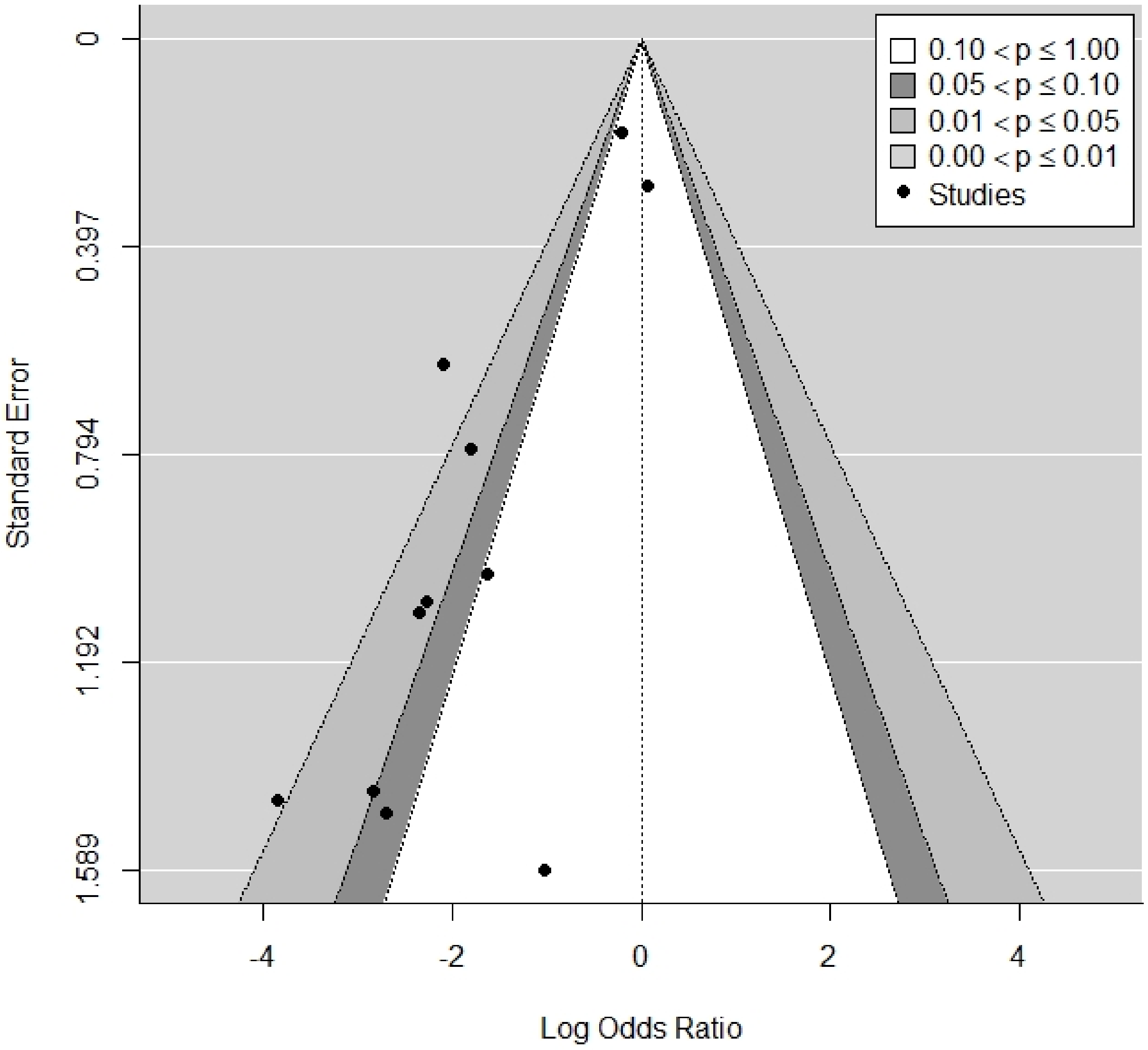

Figure S11. Traffic light plot to illustrate risk of bias evaluation of the only RCT included according to the RoB 2 framework.

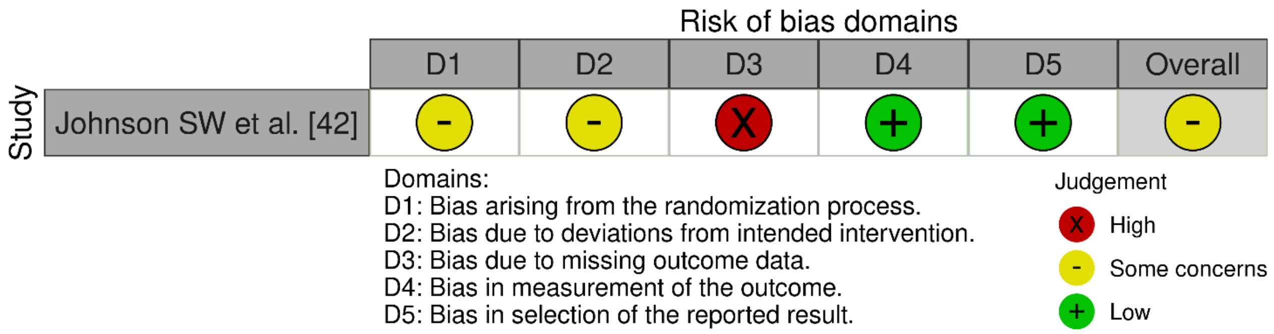

Abbreviations: RCT, randomized controlled trial; RoB, risk of bias.

Table S4 (a). Definition for the adapted version of the Newcastle-Ottawa Assessment Scale used to the purposes of the present review

	1	2	3	4	5	6	7	8	9	10	11	12	Total
Carignan A et al. [33]	2	2	0	2	1	2	2	2	2	2	2	2	19
Van Hise et al. [34]	2	2	0	2	1	2	2	2	2	2	2	2	18
Papic N et al. [35]	2	2	0	2	1	2	2	2	1	2	2	2	18
Splinter LE et al. [36]	2	2	0	2	1	2	2	2	2	2	2	2	19
Bajrovic V et al. [37]	2	2	0	2	1	2	2	2	1	2	2	2	18
Caroff DA et al. [38]	2	2	0	2	1	2	2	2	2	2	2	2	19
Ganetsky A et al. [39]	2	2	0	2	1	2	2	2	1	2	2	2	18
Knight EM et al. [40]	2	2	0	2	1	2	2	2	1	2	2	2	18
Morrisette T et al. [41]	2	2	0	2	1	2	2	2	2	2	2	2	19
Bao H et al. [43]	2	2	0	2	1	2	2	2	2	2	2	2	19

Table S4 (b). Definitions for methodological index for non-randomized studies (MINORS) used to the purposes of the present review.

Methodological items	
1	*A clearly stated aim*:<br>• the question addressed should be precise and relevant in the light of available literature.
2	*Inclusion of consecutive patients*:<br>• all patients potentially fit for inclusion (satisfying the criteria for inclusion) have been included in the study during the study period (no exclusion or details about the reasons for exclusion.
3	*Prospective collection of data:*<br>• data were collected according to a protocol established before the beginning of the study.
4	*Endpoints appropriate to the aim of the study:*<br>• unambiguous explanation of the criteria used to evaluate the main outcome which should be in accordance with the question addressed by the study. Also, the endpoints should be assessed on an intention-to-treat basis.
5	*Unbiased assessment of the study endpoint:*<br>• blind evaluation of objective endpoints and double-blind evaluation of subjective endpoints. Otherwise the reasons for not blinding should be stated.
6	*Follow-up period appropriate to the aim of the study:*<br>• the follow-up should be sufficiently long to allow the assessment of the main endpoint and possible adverse events.
7	*Loss to follow up less than 5%:*<br>• all patients should be included in the follow up. Otherwise, the proportion lost to follow up should not exceed the proportion experiencing the major endpoint.
8	*Prospective calculation of the study size:*<br>• information of the size of detectable difference of interest with a calculation of 95% confidence interval, according to the expected incidence of the outcome event, and information about the level for statistical significance and estimates of power when comparing the outcomes.
9	*An adequate control group:*<br>• having a gold standard diagnostic test or therapeutic intervention recognized as the optimal intervention according to the available published data.
10	*Contemporary groups:*<br>• control and studied group should be managed during the same time period (no historical comparison).
11	*Baseline equivalence of groups:*<br>• the groups should be similar regarding the criteria other than the studied endpoints. Absence of confounding factors that could bias the interpretation of the results.
12	*Adequate statistical analyses:*<br>• whether the statistics were in accordance with the type of study with calculation of confidence intervals or relative risk.

The items are scored 0 (not reported), 1 (reported but inadequate) or 2 (reported and adequate). The global ideal score being and 24 for comparative studies.

Table S5. Certainty of evidence according to the GRADE framework.

Certainty assessment							№ of patients		Effect		Certainty	Importance
**№ of studies**	**Study design**	**Risk of bias**	**Inconsistency**	**Indirectness**	**Imprecision**	**Other considerations**	**OVP**	**No OVP**	**Relative (95% CI)**	**Absolute (95% CI)**		
**CDI occurrence**												
11	observational studies (10) plus one randomized controlled trial	serious[a]	serious[b]	not serious	not serious	publication bias strongly suspected; very strong association; dose-response gradient[c]	104/929 (11.2%)	300/2011 (14.9%)	**OR 0.13** (0.04 to 0.38)	**127 fewer per 1.000** (from 142 fewer to 87 fewer)	⊕⊕◯◯ Low	CRITICAL (primary outcome)
**VRE infections**												
5	observational studies	serious[a]	serious[b]	not serious	not serious	publication bias strongly suspected[d]	6/255 (2.4%)	7/741 (0.9%)	**RD 0.00** (-0.03 to 0.02)	**0 fewer per 1.000** (from 0 fewer - to 0 fewer)	⊕◯◯◯ Very low	IMPORTANT (secondary outcome)

Abbreviations: CI, confidence interval; OVP: oral vancomycin prophylaxis; OR, odds ratio; RD, risk difference; VRE: vancomycin-resistant *Enterococci*.

Explanations

a. Moderate risk of bias according to MINORS tool as for observational studies.

b. Relevant differences existing about OVP doses, duration as well as population and follow-up.

c. Publication bias suspected on the basis of the funnel plot and on the Egger's test. Reasons to rate up evidence are very large effect (OR < 0.2) and dose-response gradient in the light of larger effect size when OVP duration increases.

d. Publication bias not directly assessed but inferred from the primary outcome.

GRADE Definitions about certainty of evidence.
High: Further research is very unlikely to change our confidence in the estimate of effect.
Moderate: Further research is likely to have an important impact on our confidence in the estimate of effect and may change the estimate.
Low: Further research is very likely to have an important impact on our confidence in the estimate of effect and is likely to change the estimate.
Very low: Any estimate of effect is very uncertain.

.
